# Antiangiogenesis Efficacy of Ethanol Extract from *Amomum tsaoko* in Ovarian Cancer through Inducing ER Stress to Suppress p-STAT3/NF-kB/IL-6 and VEGF Loop

**DOI:** 10.1155/2020/2390125

**Published:** 2020-02-29

**Authors:** Cheng Chen, Fei You, FengHua Wu, YuShen Luo, GuoHua zheng, HanLin Xu, Yi Liu

**Affiliations:** Department of Medicinal Chemistry, School of Pharmacy, Hubei University of Chinese Medicine, Wuhan 430065, China

## Abstract

Natural plants are considered as a huge treasure for anticancer. *Amomum tsaoko*, a plant of Zingiberaceae, is used widely as a food and traditional medicine in East Asia. In previous studies, *Amomum tsaoko* has antitumor effect on liver cancer cells, but the mechanism is not clear. Here, we demonstrated that ethanol extract from *Amomum tsaoko* (At-EE) could inhibit ovarian cancer and decrease angiogenesis *in vivo*. At-EE did not influence vascular endothelial cells directly, but decreased IL-6 and VEGF secreted by ovarian cancer cells to inhibit angiogenesis through inhibition of p-STAT3 and NF-kB activation. In addition, we demonstrated that p-STAT3 and NF-kB could adjust each other and IL-6 and VEGF also mediate p-STAT3 and NF-kB too, which created a loop. In addition, At-EE interrupted p-STAT3/NF-kB/IL-6 and VEGF loop through induced ER stress. These results reveal that p-STAT3/NF-kB/IL-6 and VEGF is a cascade amplification loop in ovarian cancer for angiogenesis, and induced ER stress can interrupt it. Taken together, this work explored the anticancer activities of *Amomum tsaoko*, which could be a potential therapeutic candidate in the treatment of ovarian cancer.

## 1. Introduction

Ovarian cancer has high mortality rate among all gynecological tumors in the United States [1]. Cytoreductive surgery in cooperation with systemic chemotherapy is the standard treatment for ovarian cancer in current. Although detection methods and treatment methods have been developed greatly, the mortality rate of ovarian cancer did not significantly decrease during the last 30 years [2]. Among the reasons for this, poor understanding of ovarian tumor biology, drug resistance, and side effect of current chemical drugs are the most critical.

Angiogenesis is a key factor during tumor growth, and many factors are involved in it such as cytokines, changes in gene expression, and tumor microenvironment. In ovarian cancer, peritoneal dissemination is the main mode, but hematogenous metastasis is proven as another important way in a recent study [3]. Targeting angiogenesis is a well-established approach in cancer therapy [4]. But drugs for antiangiogenesis usually have many limitations such as side effects and high cost. So it is important to screen drugs for antiangiogenesis with low cost and side effects. In recent years, more and more researchers focus on naturally occurring plant sources which have hypotoxicity, targeting, and therapeutic effects on types of cancers [5].

IL-6 and VEGF have been demonstrated for importance in growth, metastasis, and progression in many tumors [6, 7]. In ovarian cancer, IL-6 and VEGF are critical cytokines that are largely secreted in tumor tissue and ascites [8, 9] and are associated with many worse prognosis factors in ovarian cancer [10, 11], which take part in angiogenesis. STAT3, a member of STAT family, modulates the transcription of genes, which can regulate cell survival, proliferation, metastasis, angiogenesis, autophagy, drug resistance, and immune responses [12, 13]. Many cytokines and growth factors, especially IL-6, can activate STAT3 [14]. Interestingly, inhibition of STAT3 activation can decrease the expression of IL-6 [13]. These hint that IL-6 and p-STAT3 can regulate each other. Nuclear factor kB (NF-kB) is a nuclear transcription factor, which can regulate apoptosis, drug resistance, metastasis, inflammation, and immune responses and is thought to be a regulator for the secretion of cytokines [15, 16]. p-STAT3 and NF-kB can adjust each other in different conditions [17, 18]. But the relationship and the effect on angiogenesis of p-STAT3, NF-kB, IL-6, and VEGF are still obscure.

Plants of *Zingiberaceae* have been proven to have a good anticancer effect in many types of tumors [19–22] and are considered as excellent resources for searching natural products by more and more scientists. *Amomum tsaoko*, a plant of *Zingiberaceae*, is a food and traditional Chinese medicine having anti-inflammatory effect against insects and antitumor effect in liver cancer cells [23–25]. In this study, we demonstrate that *Amomum tsaoko* has good antitumor effect on ovarian cancer. And p-STAT3/NF-kB/IL-6 and VEGF is a cascade amplification loop in ovarian cancer. *Amomum tsaoko*-induced ER stress interrupts p-STAT3/NF-kB/IL-6 and VEGF loop to inhibit angiogenesis. Meanwhile, *Amomum tsaoko* does not influence normal cells at high concentration. Our data will help to find the effective substances and provide assistance for *Amomum tsaoko's* subsequent study.

## 2. Materials and Methods

### 2.1. Reagents and Cells

The antibodies for CD31, phospho-STAT3 (Tyr705), NF-kB (p65), GRP78, CHOP, and glyceraldehyde-3 phosphate dehydrogenase (GAPDH) were bought from Cell Signaling Technology. The antibodies for horseradish peroxidase-conjugated anti-mouse IgG and anti-rabbit IgG were bought from Epitomics. Stattic and PDTC were bought from Sigma. ELISA IL-6 kit and VEGF kit were purchased from R&D Systems. An Annexin V-FITC apoptosis kit was purchased from KeyGEN. SKOV3 cells from American Type Cell Culture (ATCC, Manassas, VA, USA) were grown in DMEM supplemented with 10% fetal bovine serum (FBS), 100 U/ml penicillin, 100 *μ*g/ml streptomycin, and 25 *μ*g/ml amphotericin B. Cells were cultured at 37°C in a humidified incubator with 5% CO_2_ and 95% air.

### 2.2. Preparation of Extract

*Amomum tsaoko* fruits cultivated in Yunnan, China, were purchased from an herbal medicine store in Wuhan, China. *Amomum tsaoko* was pulverized by using a blender. The powder (1 kg) was refluxed in 3 L of 75% ethanol at 100°C for 8 h. The supernatant solution was filtered through Whatman filter paper #2, after which the filtrate was evaporated in a rotary vacuum evaporator and subsequently freeze-dried at −70°C. The resulting powder was used as an ethanol extract of At (At-EE) and preserved at −20°C for use. The yield of At-EE was 198.6 g per kilogram of dried powder.

### 2.3. MTT Assay

The MTT assay was done to evaluate the function of At-EE on the proliferation of ovarian tumor cells. In 96-well plates, 1 × 10^4^ cells were seeded into a well with 200 *μ*l medium. Then, various concentrations of At-EE were added into each well. 48 h later, the MTT solution was added into each well and incubated for 4 h at 37°C in a humidified incubator with 5% CO_2_ and 95% air. Each well was measured by enzyme-labeled instrument (Thermo) at 570 nm absorbance.

### 2.4. Annexin V-FITC-Propidium Iodide Assay

Cells were grown in 12-well plates and reached over 80% confluence. Then, cells were treated with different concentrations of At-EE. At the indicated time later, the cells were washed twice with PBS and harvested and resuspended with 500 *μ*l binding buffer in Eppendorf tube. Next, 5 *μ*l Annexin V-FITC and 10 *μ*l propidium iodide were added into the Eppendorf tube for incubation in the dark for 10 min at 4°C. 10,000 events per sample were analyzed and calculated with a FACS Calibur flow cytometer (BD Biosciences, Franklin Lakes, NJ, USA) using WinMDI 2.8 software.

### 2.5. Wound Healing Assays

At first, ovarian cells were cultured at 95% confluence, which were scratched with a 200 *μ*l tip, washed with cold PBS, and grown in DMEM supplemented with 1% FBS (treated with DMSO or At-EE for 24 h). The same fields of the scratched margin were photographed at 0 and 24 h. The percent wound closure was calculated as the ratio of the unclosed area at 24 h to the wounded area at 0 h (set to 100%).

### 2.6. Invasion Assay

Boyden chemotaxis chamber was used for cell invasion assays. Ovarian cells were pretreated with DMSO or At-EE for 24 h. 50 *μ*l of suspended cells (1 × 10^5^ cells/ml) in serum-free media was placed into the upper chamber, and the complete growth medium was placed in the lower chamber. After incubation for 24 h, the cells on the upper surface of the filter were wiped with a swab. The cells on the lower surface of the filters were fixed with paraformaldehyde for 10 min and then stained with crystal violet for 1 h. The number of cells on the lower surface of the filter was counted by microscopy.

### 2.7. In Vitro Angiogenesis Evaluation

24-well plates were coated with cold Matrigel (BD Biosciences). Then, 1 × 10^5^ HUVEC cells suspended in the conditioned media from ovarian cells treated by DMSO or At-EE were seeded in Matrigel-coated well. 16 hours later, the number of capillary-like tubes was counted by using microscopy in five randomly selected fields, and the average was calculated.

### 2.8. Western Blot Analysis

Cells were collected and lysed in RIPA buffer (50 mM Tris-HCl, 1 mM EDTA, 150 mM NaCl, and 1% NP-40) containing 1 mM PMSF and a cocktail of protease inhibitors. After 30 min on ice, the mixtures were centrifuged at 12,000 rpm for 15 min, and the supernatant was harvested. The concentration of each sample was determined by using the Bio-Rad protein assay reagent. 50 *μ*g of total protein from each sample was separated by SDS-PAGE gel and then was electrophoretically transferred to a PVDF membrane. After the transfer, membranes were blocked with TBS containing 5% nonfat dry milk at room temperature for 1 h. Then, the membranes were incubated with the primary antibody at 4°C overnight, followed by incubation with the HRP-linked secondary antibody. Finally, the immune bands were revealed via fluorography using an enhanced ECL system.

### 2.9. Enzyme-Linked Immunosorbent Assay

Concentrations of IL-6 and VEGF were detected by ELISA kits from R&D Systems according to the manufacturers' protocol.

### 2.10. In Vivo Experiments

This study was performed with approval from the Committee on the Ethics of Animal Experiments in the Hubei province. All animal experiments were carried out in accordance with the Guide for the Care and Use of Laboratory Animals of Tongji Hospital in Hubei.

SKOV3 cells (3 × 10^6^ in 100 *μ*l of PBS) were injected into the ovarian bursa of BALB/c nude mice. 7 days later, the mice were randomized into two groups (eight mice/group) and treated with 30 mg/kg At-EE or a vehicle control injected with the same volume of saline for oral administration, three times a week, 4 weeks. 4 weeks later, cancer cells in the abdominal cavity were evaluated by *in vivo* bioluminescence imaging. After the experiment, the mice were sacrificed, and the tumors were resected.

SKOV3 cells (5 × 10^6^ in 100 *μ*l of PBS) were injected subcutaneously into the right suprascapular region of mice. Tumor volume was estimated by using the following formula: volume = length × width^2^/2. About 1 week after tumor implantation, when the tumor reached a mean group size of 50 mm^3^, the mice were randomized into two groups (eight mice/group) and treated with 30 mg/kg At-EE or a vehicle control injected with the same volume of saline for oral administration, three times a week, 5 weeks. The tumor volumes were determined by caliper measurement once a week.

### 2.11. Immunohistochemical Staining

Tumors were stored in 10% formalin over 24 h and then embedded in paraffin and sectioned. The expressions of CHOP, p-STAT3, NF-kB, VEGF, and IL-6 were detected by streptavidin-peroxidase way.

### 2.12. Statistical Analysis

The data were presented as means ± standard deviation (SD). All experiments were done at least three times and evaluated by one-way ANOVA. Statistical analysis was performed using the SPSS 22.0. *p* < 0.05 was considered statistically significant.

## 3. Result

### 3.1. At-EE Had Antitumor Efficacy in Ovarian Cancer

**Figure 1:**
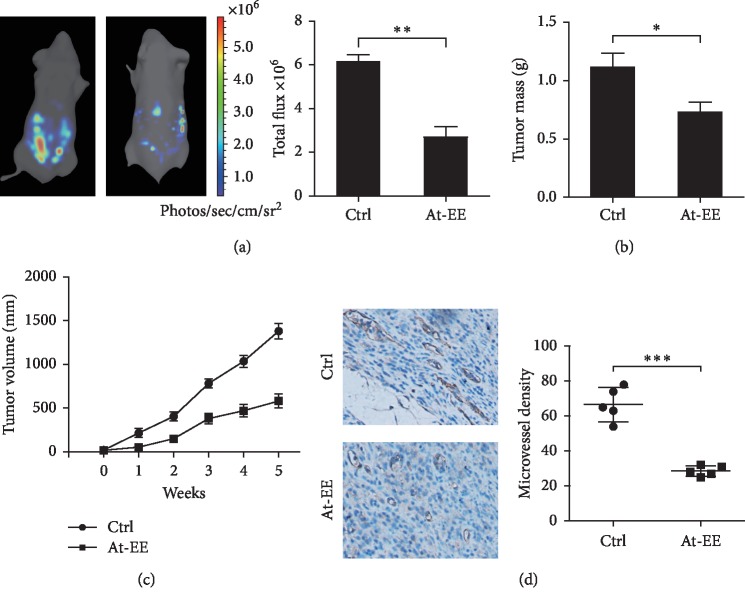
At-EE had antitumor efficacy in ovarian cancer. (a, b) 3 × 10^6^ cells were injected into the ovarian bursa of BALB/c nude mice, which were randomized into control group and At-EE group. (a) 4 weeks later, cancer cells in the abdominal cavity were counted by *in vivo* bioluminescence imaging. (b) After the experiment, the mice were sacrificed, and the tumors were resected. (c) 5 × 10^6^ cells were inoculated into ovarian bursa of BALB/c nude mice, which were randomized into control group and At-EE group. (d) The expression of CD31 was detected by immunohistochemical staining. After finishing the experiment, tumor tissues from each mouse were resected for measurement. The results were similar in at least three independent experiments. ^*∗*^*p* < 0.05. ^*∗∗*^*p* < 0.01.

To investigate the anticancer effect of At-EE in ovarian cancer, models of tumor-bearing mice were established. First, SKOV3 cells (3 × 10^6^ in 100 *μ*l of PBS) were injected into the ovarian bursa of BALB/c nude mice. 5 weeks later, the result was detected. As shown in Figure 1(a), luciferase activity was declined in the At-EE treatment group compared with the control group. And the tumor masses of the At-EE treatment group were remarkably lower than those of the control group (Figure 1(b)). Second, SKOV3 cells (5 × 10^6^ in 100 *μ*l of PBS) were injected s.c. into the right suprascapular region of BALB/c nude mice. We obtained the same result in a time course in this model (Figure 1(c)). After measuring the last tumor, the mice were sacrificed, and the tumor masses from tumor-bearing mice were obtained and analyzed by immunohistochemistry. As shown in Figure 1(d), the expression of CD31 in the At-EE treatment group was significantly lower than that in the control group.

These results indicated that At-EE has good antitumor potential in ovarian cancer and inhibited the angiogenesis in this process.

### 3.2. At-EE Decreased Ovarian Cancer-Mediated Angiogenesis

**Figure 2:**
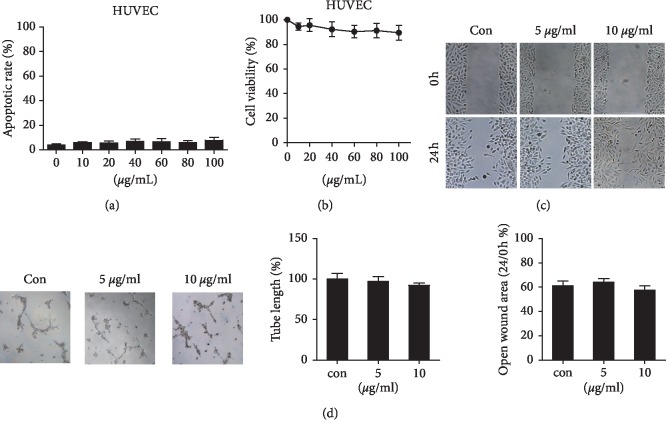
At-EE did not influence HUVEC cells directly. (a–d) HUVEC cells were treated with DMSO or At-EE. (a) The apoptotic rate was assessed by flow cytometry. (b) The cell viability rate was analyzed by MTT assay. (c) Wound healing assays were done for the mobility of HUVEC cells. (d) In vitro angiogenesis evaluation was done for HUVECs treated by At-EE. The results were similar in at least three independent experiments. ^*∗*^*p* < 0.05. ^*∗∗*^*p* < 0.01.

**Figure 3:**
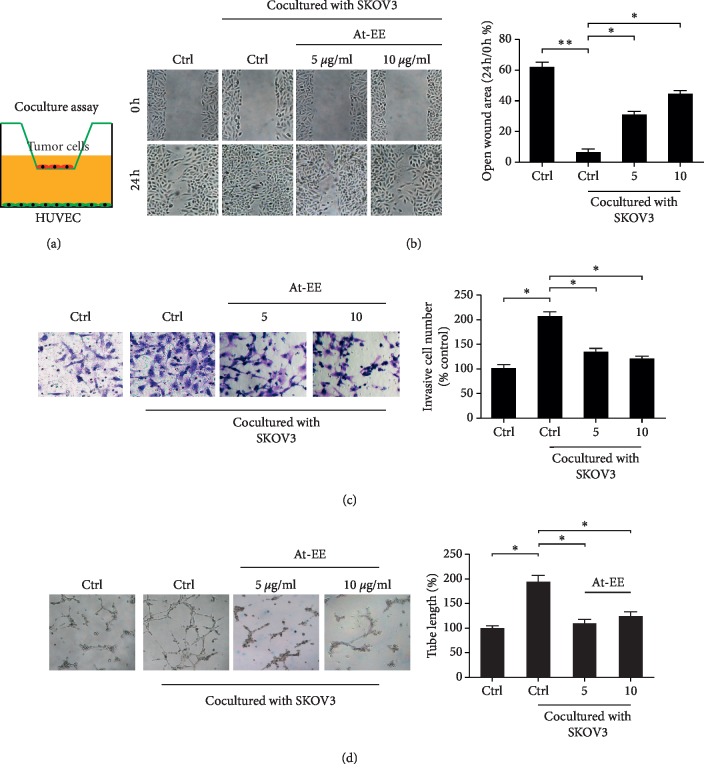
At-EE decreased ovarian cancer-mediated angiogenesis. (a–d) HUVEC cells were treated by condition media from ovarian cancer cells treated by At-EE or DMSO. (a) Representative diagram of the coculture assay. (b) Wound healing assays were done for the mobility of HUVEC cells. (c) Cell invasion assays were done for metastasis of HUVEC cells. (d) In vitro angiogenesis evaluation was done for HUVEC cells. The results were similar in at least three independent experiments. ^*∗*^*p* < 0.05. ^*∗∗*^*p* < 0.01.

As mentioned before, peritoneal dissemination is considered as the main way of ovarian cancer metastasis. But hematogenous metastasis is proven as another important method in ovarian cancer in a recent study [3]. And angiogenesis is a hallmark in ovarian cancer, which benefits to cell growth and metastasis [26]. We wanted to know how At-EE inhibited angiogenesis. At first, we focused on whether At-EE affected endothelial cells directly. As shown in Figures 2(a), 2(b), and [Supplementary-material supplementary-material-1], At-EE did not induce apoptosis and affect proliferation in HUVEC cells and immortalized human keratinocyte cell line HaCaT. And At-EE also did not influence the migratory and angiogenesis of capabilities of HUVEC cells (Figures 2(c) and 2(d)). From the above result, we obtained a conclusion that At-EE did not directly affect vascular endothelial cells and normal cells. So we used a coculture assay to find whether At-EE could impact the feedback between endothelial cells and tumor cells. As described in the previous study [27], cells were in the upper chamber while HUVEC cells were in the lower wells (Figure 3(a)). These two chambers were separated by a membrane, and only secreted factors can transfer from it. In would healing assay, the migration of HUVEC cells was increased in cocultured with the SKOV3 group, and At-EE could partially reverse this effect (Figure 3(b)). Further, the effect of At-EE on invasion of HUVEC cells was evaluated. As shown in Figure 3(c), the invasion ability of HUVEC cells was increased in cocultured with the medium of SKOV3 cells. And At-EE could notably suppress this phenomenon. We obtained the same results in endothelial tube formation assays (Figure 3(d)). All these results suggested that At-EE may affect the movement and formation of vascular endothelial cells by regulating tumor cell-secreted cytokines.

### 3.3. At-EE Decreased Expression of VEGF and IL-6 from Ovarian Cancer to Inhibit Angiogenesis

Various cytokines are considered as benefiting angiogenesis. We wanted to measure the mRNA expressions of these cytokines between SKOV3 cells and SKOV3 cells treated with At-EE. At first, we evaluated the apoptotic effect and the antiproliferative effects of At-EE on SKOV3 cells to find the appropriate concentration. As shown in Supplementary [Supplementary-material supplementary-material-1], At-EE significantly increased apoptosis rate and inhibited proliferation effects of SKOV3 over 10 *μ*g/ml concentration. So we chose the concentration range of 0–10 *μ*g/ml in the following assays to evaluate the antiangiogenesis effect of At-EE.

**Figure 4:**
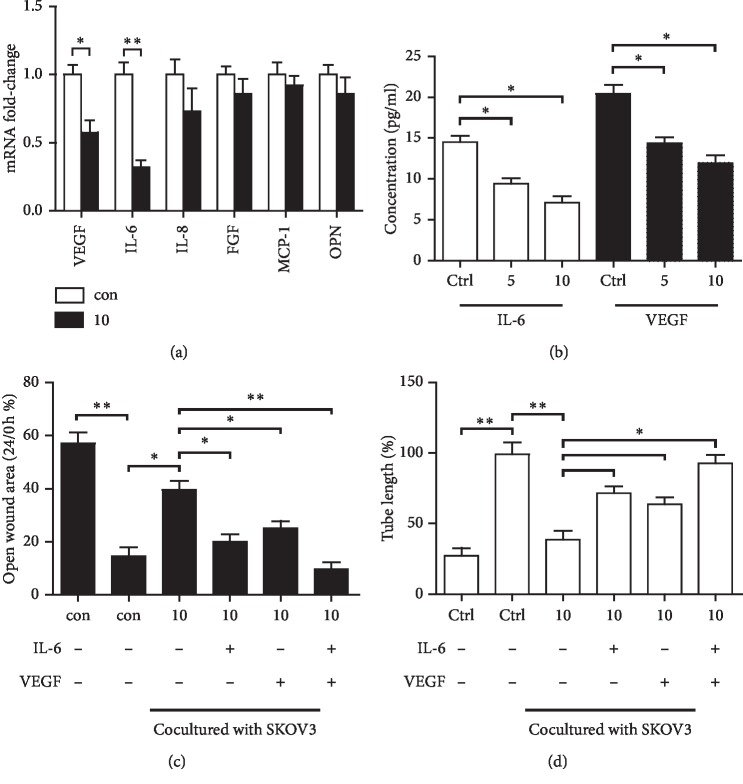
At-EE decreased IL-6 and VEGF production of ovarian cancer to inhibit angiogenesis. (a) SKOV3 was treated by At-EE for 48 h. Then, the mRNAs of IL-8, VEGF, FGF, MCP-1, OPN, and IL-6 were detected by real-time PCR. (b) SKOV3 was treated by At-EE for 48 h. Then, IL-6 and VEGF in conditioned media were detected by ELISA kits. (c) Wound healing assays were done for the mobility of HUVECs treated by condition media from ovarian cancer cells treated by At-EE in the presence or absence of IL-6, VEGF, or IL-6 and VEGF. (d) In vitro angiogenesis evaluation was done for HUVECs treated by condition media from ovarian cancer cells treated by At-EE in the presence or absence of IL-6, VEGF, or IL-6 and VEGF. The results were similar in at least three independent experiments. ^*∗*^*p* < 0.05. ^*∗∗*^*p* < 0.01.

The mRNA expressions of VEGF, IL-8, and IL-6 were decreased in the At-EE treatment group, but only VEGF and IL-6 had significant difference in the SKOV3 group compared with the At-EE treatment group (Figure 4(a)). Next, VEGF and IL-6 were detected by ELISA in the culture medium from these two groups. As shown in Figure 4(b), VEGF and IL-6 were lower in the medium from the At-EE treatment group at 5 and 10 *μ*g/ml concentration. In addition, adding IL-6 or VEGF into the culture medium of HUVEC cells reversed the ability of At-EE inhibiting angiogenesis, and the effect of adding IL-6 and VEGF at the same time was better than that of adding IL-6 or VEGF (Figures 4(c) and 4(d)). These pieces of evidences demonstrated VEGF and IL-6 secreted by ovarian cancer cells were the key factors for angiogenesis, and At-EE could inhibit them.

### 3.4. At-EE Interrupted p-STAT3/NF-kB/IL-6 and VEGF Loop to Inhibit Ovarian Cancer-Induced Angiogenesis

**Figure 5:**
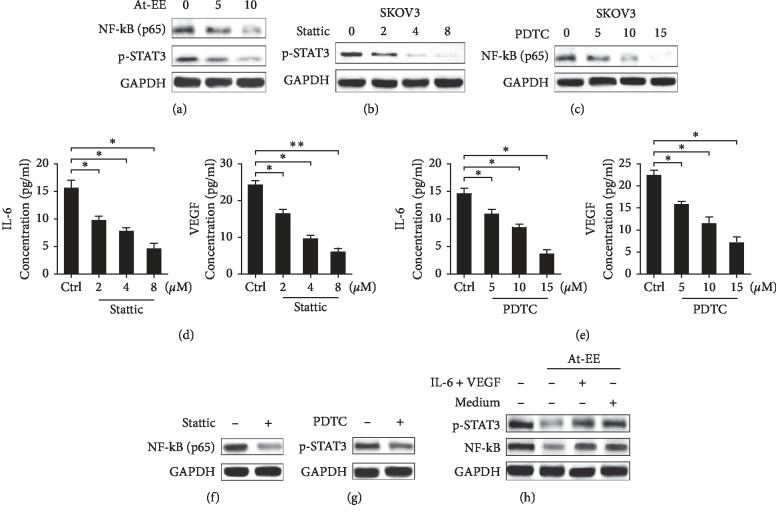
At-EE suppressed NF-kB/p-STAT3/IL-6 and VEGF loop. (a) Cells were treated with At-EE at 5 or 10 *μ*g/ml for 48 h, and NF-kB and p-STAT3 were analyzed by western blot. (b) Cells were incubated with stattic at 2, 4, or 8 *μ*M for 48 h, and p-STAT3 was analyzed by western blot. (c) Cells were incubated with PDTC at 5, 10, or 15 *μ*M for 48 h, and NF-kB was analyzed by western blot. (d) SKOV3 was treated by stattic for 48 h. Then, IL-6 and VEGF in conditioned media were detected by ELISA kits. (e) SKOV3 was treated by PDTC for 48 h. Then, IL-6 and VEGF in conditioned media were detected by ELISA kits. (f) Cells were incubated with stattic, and NF-kB was analyzed by western blot. (g) Cells were incubated with PDTC, and p-STAT3 was analyzed by western blot. (h) Cells were treated with condition media form ovarian cancer cells treated by At-EE in presence or absence of IL-6 and VEGF, and NF-*k*B and p-STAT3 were analyzed by western blot. The results were similar in at least three independent experiments. ^*∗*^*p* < 0.05. ^*∗∗*^*p* < 0.01.

NF-kB is proven as a cytokine promoter [15, 16] and is constitutively active in many tumors [28]. Activation of STAT3, the upstream gene of NF-kB, is demonstrated to take part in promoting proliferation, drug resistance, and metastasis in ovarian cancer [13, 29]. So we obtained a hypothesis that At-EE might influence p-STAT3/NF-kB axis-induced IL-6 and VEGF. As we assume, At-EE inhibited NF-kB and p-STAT3 activation over 5 *μ*g/ml concentration in a dose-dependent manner (Figure 5(a)). We used PDTC (pyrrolidine dithiocarbamate, a NF-kB special inhibitor) and stattic (a p-STAT3 inhibitor) to evaluate whether NF-kB or p-STAT3 mediates IL-6 and VEGF production and participating angiogenesis. As shown in Figures 5(b) and 5(c), NF-kB activation of SKOV3 cells was significantly inhibited by PDTC and STAT3 activation was inhibited by stattic. And IL-6 and VEGF were decreased by PDTC or stattic in a dose-dependent manner (Figures 5(d) and 5(e)). In addition, we demonstrated stattic could inhibit NF-kB phosphorylation, and STAT3 phosphorylation was suppressed by PDTC (Figures 5(f) and 5(g)). As shown in Figure 5(h), adding IL-6 and VEGF reversed the effect of At-EE and decreased NF-kB and p-STAT3 activation, and adding the medium of normal cultured SKOV3 cells had the same effect. Above these results proved that p-STAT3/NF-kB was a loop, and VEGF and IL-6 could feedback and increase this loop. And At-EE could interrupt the cascade amplification loop.

### 3.5. At-EE Induce ER Stress to Interrupt p-STAT3/NF-kB Loop to Inhibit Ovarian Cancer-Induced Angiogenesis

**Figure 6:**
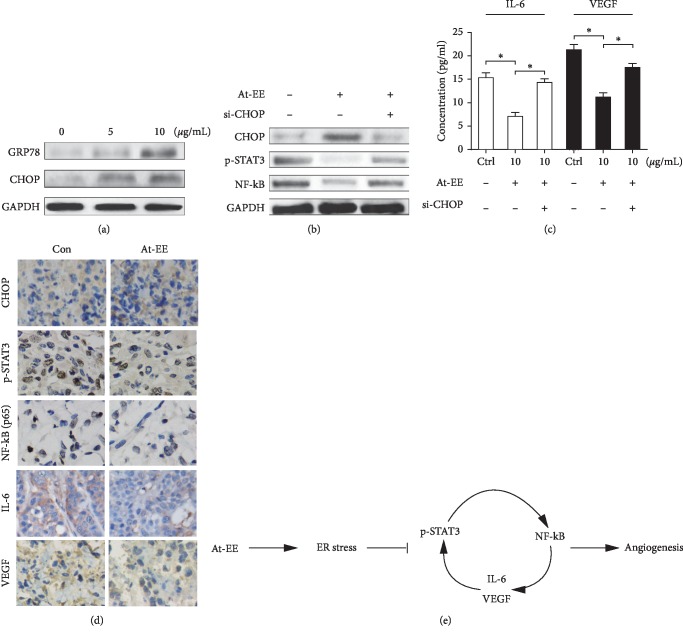
At-EE induced ER stress to suppress NF-kB/p-STAT3/IL-6 and VEGF loop. (a) Cells were treated with At-EE at 5 or 10 *μ*g/ml for 48 h, and GRP78 and CHOP were analyzed by western blot. (b) Cells or cells transfected with specific CHOP siRNA were treated by At-EE or DMSO, and NF-kB and p-STAT3 were analyzed by western blot. (c) Cells or cells transfected with specific CHOP siRNA were treated by At-EE or DMSO, and IL-6 and VEGF in conditioned media were detected by ELISA kits. (d) The expressions of p-STAT3, NF-kB, CHOP, VEGF, and IL-6 were detected by immunohistochemical staining. (e) Schematic model illustrating the potential pathway associated with At-EE inhibiting ovarian cancer cells. The results were similar in at least three independent experiments. ^*∗*^*p* < 0.05. ^*∗∗*^*p* < 0.01.

ER stress is provoked by the accumulation of unfolded or misfolded proteins in the endoplasmic reticulum (ER) lumen, which negatively regulates p-STAT3 in our previous studies. And ER stress took part in normal cells and cancer cells [30–32]. However, it is unclear whether *Amomum tsaoko* induces ER stress and there is a link between ERS and angiogenesis in ovarian cancer. GRP78 and CHOP are the makers of ER stress [1]. As shown in Figure 6(a), At-EE increased the expression of GRP78 and CHOP in a dose-dependent manner, which was the evidence of ER stress induced by At-EE. Then, SKOV3 cells were transfected with specific siRNAs targeting CHOP to explore whether ER stress contributes to At-EE interrupted p-STAT3/NF-kB/IL-6 and VEGF loop. As expected, the At-EE-induced upregulation of CHOP was abrogated in cells expressing CHOP siRNAs, and downregulation of CHOP restored the protein expression levels of phosphorylated STAT3 and NF-kB (Figure 6(b)). In addition, notably preventing CHOP elevation restored the IL-6 and VEGF protein levels by At-EE treatment (Figure 6(c)). The tumor masses from tumor-bearing mice (the same mice with Figures 1(c) and 1(d)) were obtained and analyzed by immunohistochemistry. As shown in Figure 6(d), the expressions of IL-6, VEGF, p-STAT3, and NF-kB were inhibited and CHOP was increased in the At-EE group compared with the control group. Altogether, above these results proved that p-STAT3/NF-kB was a crucial axis in the development of ovarian cancer for increasing the expression of IL-6 and VEGF which created a feedback loop to activate p-STAT3/NF-kB axis as a cascade amplification method, and At-EE could restrain this phenomenon.

## 4. Discussion

Recurrence, metastasis, and drug resistance are the main features of ovarian cancer [33]. Most chemotherapeutic drugs are toxic and easy to relapse. Natural plants are considered as a huge treasure for many diseases, especially anticancer. In recent studies, more and more researchers focus on plants of *Zingiberaceae*, such as *Kaempferia galanga*, *Curcuma caesia*, *Curcuma longa*, and *Alpinia officinarum*, which are proven to have good anticancer effects in many cancers [34–36]. In this study, we focused on *Amomum tsaoko*, a food and traditional Chinese medicine, which is proven to have anticancer effect on liver cancer cells [25]. And *Amomum tsaoko* often appears in ancient prescription of traditional Chinese medicine for gynecological disease, so we hypothesized whether *Amomum tsaoko* has the ability of inhibiting ovarian cancer. In this study, we obtained the important findings: (i) *in vitro* and *vivo*, we demonstrated *Amomum tsaoko* inhibited ovarian cancer in the first time through antiangiogenesis; (ii) p-STAT3/NF-kB is an axis to induce the expression of IL-6 and VEGF which created a feedback to activate p-STAT3/NF-kB axis as a cascade amplification method; and (iii) At-EE induces ER stress to interrupt the cascade amplification effect.

Results of tumor-bearing mice models demonstrated At-EE could inhibit ovarian cancer. And the expression of CD31 was lower in the At-EE group than in the control group which hinted At-EE restrained ovarian tumor through antiangiogenesis. Angiogenesis is a hallmark of ovarian cancer [37]. Apart from peritoneal dissemination, hematogenous metastasis is confirmed as an important method in ovarian cancer in recent years [3]. Cytokines derived from tumor cells are one of the important reasons for angiogenesis [38]. Many antiangiogenesis agents are approved for tumor therapy including ovarian cancer [39], but meanwhile, cause side effects [39]. Experiments of apoptosis and cell viability showed that concentrations over 20 *μ*g/ml were the effective concentrations of At-EE inhibiting ovarian tumor. But At-EE did not significantly influence immortalized human keratinocyte cell line HaCaT and HUVEC cell line within 100 *μ*g/ml concentration, which is the evidence for *Amomum tsaoko*, a great potential anticancer resource. Within 10 *μ*g/ml is the reasonable concentration to evaluate the effect of At-EE on antiangiogenesis and to avoid the effect of At-EE against tumor cells. In our study, At-EE inhibited ovarian cancer-induced angiogenesis, but did not influence HUVEC directly, which suggested that At-EE might mediate secretion of cytokines from cancer cells to inhibit angiogenesis. Through scanning the cytokine benefits for angiogenesis, we found that At-EE treatment could significantly decrease ovarian cancer cell-secreted IL-6 and VEGF. IL-6 is a crucial cytokine in tumor progression and is demonstrated as a prognostic marker for monitoring ovarian cancer [40, 41]. And IL-6/IL-6R axis is a potential target for ovarian cancer therapy [42]. VEGF is benefit to metastasis, growth, recurrent, and mediated immune cells [43–45] and is also a prognostic marker for monitoring ovarian cancer [46]. Adding IL-6 and VEGF into culture medium could reverse the ability of At-EE inhibiting angiogenesis, which confirmed At-EE restrained angiogenesis through decreasing tumor cell-secreted IL-6 and VEGF.

In the previous study, *Amomum tsaoko* has been proven to have anti-inflammatory effect. NF-kB is a nuclear transcription factor that is considered as a generator of inflammatory cytokines. NF-kB is activated by growth factors, cytokines, and stress [15, 16], and is benefit to angiogenesis [47]. STAT3, constitutive expression in ovarian cancer, is correlated with tumor growth [48] and is considered as the upstream gene of NF-kB. NF-kB and STAT3 form a network to mediate inflammatory in breast cancer cells [49]. We hypothesized At-EE inhibited the expression of IL-6 and VEGF from ovarian cancer cells through mediating NF-kB or STAT3. Our data demonstrated the hypothesis that At-EE decreased the activation of NF-kB and p-STAT3 over 5 *μ*g/ml. Inhibition of the activation of NF-kB or STAT3 could decrease IL-6 and VEGF production by using PDTC or stattic. Interestingly, PDTC inhibited p-STAT3 activation, and stattic restrained NF-kB phosphorylation, which was an evidence for p-STAT3 and NF-kB mediating each other in ovarian cancer as a loop. In retinal pigment epithelium/choroid organ, autocrine VEGF regulates NF-kB phosphorylation [50]. Autocrine IL-6 activates p-STAT3 in dendritic cells [51]. In our study, adding medium from cultured tumor cells or IL-6 and VEGF could abrogate the effect of At-EE inhibition of p-STAT3 activation and NF-kB phosphorylation, which demonstrated IL-6, VEGF, and p-STAT3/NF-kB created an autocrine feedback loop in ovarian cancer cells.

ER stress mediates protein translocation, protein folding, and protein posttranslational modifications, which is induced by the accumulation of unfolded or misfolded proteins in the endoplasmic reticulum (ER) lumen to reestablish ER homeostasis in cells. ER stress is found to be a new pathway leading to apoptosis [52]. In recent studies, ER stress is considered to be associated with angiogenesis [53, 54], but the mechanism is not well understood. In our previous studies, drug-induced ER stress inhibits the activation of p-STAT3 [1, 12]. So whether At-EE inhibited p-STAT3/NF-kB loop by activating ER stress? In this research, At-EE increased the expression of GRP78 and CHOP which is the evidence for ER stress induced. And inhibition of ER stress restored the effect of At-EE suppression of p-STAT3, NF-kB, IL-6, and VEGF by CHOP-specific siRNAs. This is the first time that ER stress activated has been shown to inhibit angiogenesis in ovarian cancer cells under drug treatment.

In conclusion, our data confirmed that *Amomum tsaoko*, a food and traditional Chinese medicine, has good antitumor effect on ovarian cancer. We also prove for the first time that p-STAT3/NF-kB is loop to increase the expression of IL-6 and VEGF, and IL-6 and VEGF create a feedback to p-STAT3/NF-kB loop in ovarian cancer cells. At-EE induces ER stress to break this cascade amplification effect to inhibit angiogenesis achieving the effect of inhibiting tumor growth. Our data provide favorable evidence and direction for research of *Amomum tsaoko.*

## Data Availability

The data used to support the findings of the study are available from the corresponding author upon request.

## Supplementary Materials

Supplementary Materials Figure S1: At-EE could not influence the apoptosis and cell viability of HaCaT cells. (A, B) HaCaT cells were treated with DMSO or At-EE. (A) The apoptotic rate was assessed by flow cytometry. (B) The cell viability rate was analyzed by MTT assay. Figure S2: At-EE could not influence the apoptosis and cell viability of HUVEC cells. (A, B) HUVEC cells were treated with DMSO or At-EE. (A) The apoptotic rate was assessed by flow cytometry. (B) The cell viability rate was analyzed by MTT assay.
