# Supplementary figures and images for: Antiangiogenesis Efficacy of Ethanol Extract from Amomum tsaoko in Ovarian Cancer through Inducing ER Stress to Suppress p-STAT3/NF-kB/IL-6 and VEGF Loop
Source: Evid Based Complement Alternat Med. 2020 Feb 29;2020:2390125. doi: 10.1155/2020/2390125 (PMC7066415; doi:10.1155/2020/2390125)

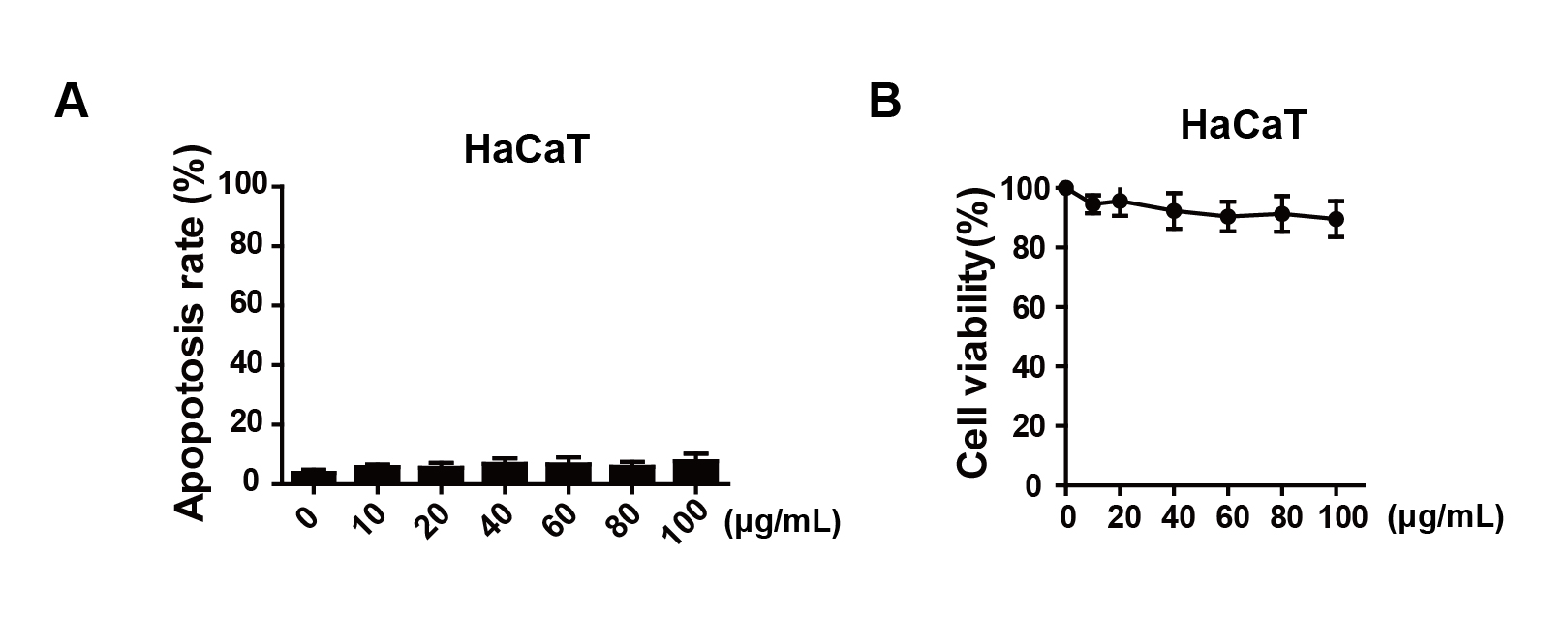

Supplement: Supplementary Materials — Figure S1: At-EE could not influence the apoptosis and cell viability of HaCaT cells. (A, B) HaCaT cells were treated with DMSO or At-EE. (A) The apoptotic rate was assessed by flow cytometry. (B) The cell viability rate was analyzed by MTT assay. Figure S2: At-EE could not influence the apoptosis and cell viability of HUVEC cells. (A, B) HUVEC cells were treated with DMSO or At-EE. (A) The apoptotic rate was assessed by flow cytometry. (B) The cell viability rate was analyzed by MTT assay. [file 2390125.f1.zip › 2390125.f1/Fig-s1.jpg]

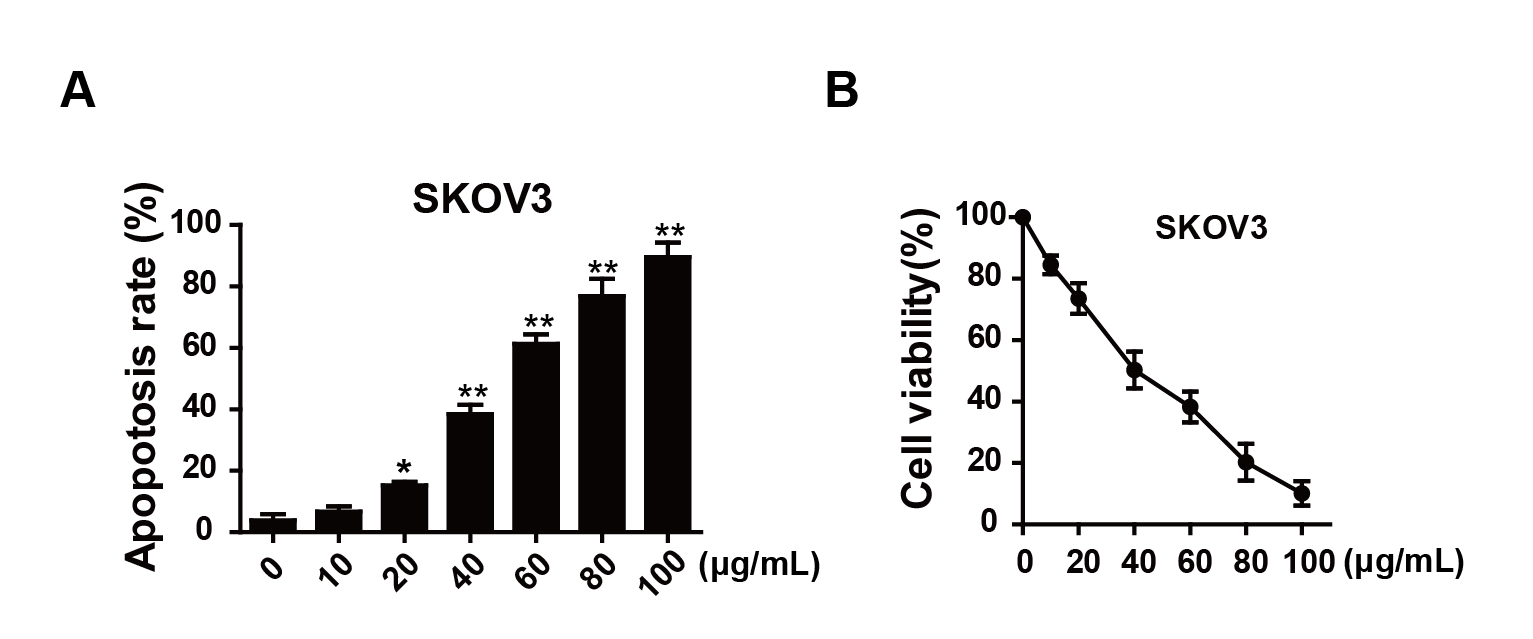

Supplement: Supplementary Materials — Figure S1: At-EE could not influence the apoptosis and cell viability of HaCaT cells. (A, B) HaCaT cells were treated with DMSO or At-EE. (A) The apoptotic rate was assessed by flow cytometry. (B) The cell viability rate was analyzed by MTT assay. Figure S2: At-EE could not influence the apoptosis and cell viability of HUVEC cells. (A, B) HUVEC cells were treated with DMSO or At-EE. (A) The apoptotic rate was assessed by flow cytometry. (B) The cell viability rate was analyzed by MTT assay. [file 2390125.f1.zip › 2390125.f1/Fig-s2.jpg]
